# An articulated Late Triassic (Norian) thalattosauroid from Alaska and ecomorphology and extinction of Thalattosauria

**DOI:** 10.1038/s41598-020-57939-2

**Published:** 2020-02-04

**Authors:** Patrick S. Druckenmiller, Neil P. Kelley, Eric T. Metz, James Baichtal

**Affiliations:** 1 University of Alaska Museum, 1962 Yukon Dr., Fairbanks, AK 99775 USA; 2 Department of Geosciences, University of Alaska Fairbanks, 900 Yukon Dr., Fairbanks, AK 99775 USA; 3 Department of Earth and Environmental Sciences, Vanderbilt University, 5726 Stevenson Center, Nashville, TN 37240 USA; 4 Department of Paleobiology, National Museum of Natural History, Smithsonian Institution, PO Box 37012, Washington, DC, 20013 USA; 5 Tongass National Forest, P.O. Box 19001, Thorne Bay, AK 99919 USA

**Keywords:** Palaeontology, Taxonomy, Palaeoecology

## Abstract

Thalattosaurians are a cosmopolitan clade of secondarily aquatic tetrapods that inhabited low-latitude, nearshore environments during the Triassic. Despite their low taxic diversity, thalattosaurians exhibit remarkable morphological disparity, particularly with respect to rostral and dental morphology. However, a paucity of well-preserved material, especially leading up to their extinction, has hampered efforts to develop a robust picture of their evolutionary trajectories during a time of profound marine ecological change. Here, we describe a new taxon based on an articulated and nearly complete skeleton from Norian sediments of southeastern Alaska, USA. The holotype is the most complete North American thalattosaurian yet described and one of the youngest occurrences of the clade worldwide. We present a new hypothesis of interrelationships for Thalattosauria and investigate potential feeding modes in the Alaskan taxon. An integrated view suggests that the absence of pelagic lifestyles and restricted ecological roles may have contributed to thalattosaurs’ eventual extinction.

## Introduction

Thalattosaurs were among several reptile lineages that adapted to marine life in the Mesozoic^1,2^. Although they obtained a circum-hemispheric distribution during their ~40 million-year existence, thalattosaurs never attained the taxonomic diversity or adaptation for open marine life observed among contemporaneous ichthyosaurs and sauropterygians, and unlike these groups did not persist into the Jurassic^3^. In contrast with their low taxic diversity, thalattosaur genera exhibit substantial anatomical disparity, particularly in dentition and rostral structure^4^, and spanned a broad range in body size (<1 m to ~5 m), suggesting they occupied a variety of ecological niches in the nearshore realm.

Despite their broad geographic distribution, thalattosaur fossils are rare, especially articulated specimens, which are known from only a few localities in the Western and Eastern Tethyan realms (modern day Europe and China)^5,6^. Although thalattosaur fossils were first discovered in North America^7^, a complete articulated specimen from the eastern margin of Panthalassa (contemporary western North America) has never been reported. Moreover, Norian-aged material, representing the youngest occurrences of this group prior to their extinction, is limited to a few records from the Alpine region of Europe^5^ and western North America^8^. Most of these occurrences are highly fragmentary, and only a single reasonably complete Norian thalattosaur, *Endennasaurus*^5^, has been previously described. This patchy geographic and temporal fossil distribution means that the origins, extinction and phylogeny of thalattosaurs, as well as their position within Diapsida remain poorly understood.

**Figure 1:**
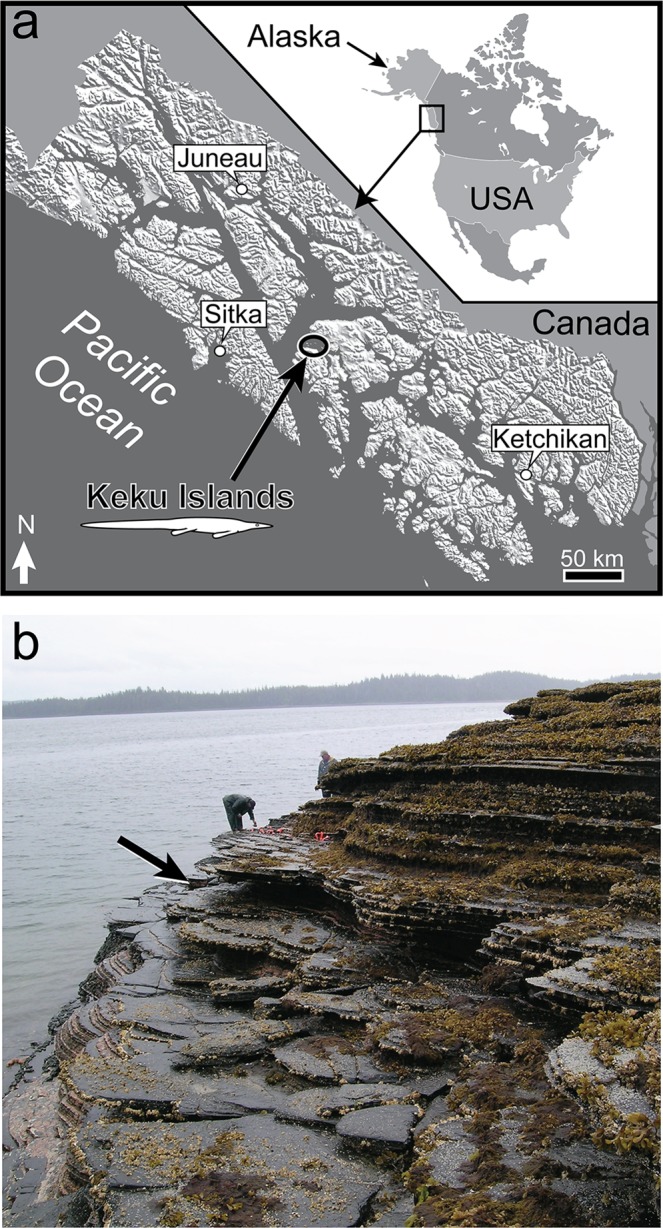
Location and collection site. (**a**) Map of southeastern Alaska showing Keku Islands (modified from Riehle *et al*.^50^). (**b**) Photograph of intertidal outcrop where UAMES 23258 was discovered (arrow). (Image courtesy Thomas Adams; used with permission).

Here we report the first articulated and substantially complete thalattosaur fossil from North America (Fig. 1). This relatively small animal exhibits numerous cranial and postcranial characteristics that distinguish it from all previously described genera. Our newly assembled and expanded phylogenetic dataset reveals the new taxon to occupy a basal position within Thalattosauria, while representing the stratigraphically youngest record of the clade in North America. The specimen expands the known morphological disparity recognized in clade, and provides valuable insight into thalattosaur feeding ecology, biogeography and extinction.

## Results

### Systematic paleontology

Thalattosauria Merriam, 1904

Thalattosauroidea Nopsca, 1928

*Gunakadeit* gen. nov.

*Gunakadeit joseeae* sp. nov.

### Etymology

The generic name is derived from *Gunakadeit*, (pronounced Goo-na’-ka-date), a sea monster of the Tlingit culture. The specific ending honors Joseé Michelle DeWaelheyns, mother of the discoverer.

### Holotype

**Figure 2:**
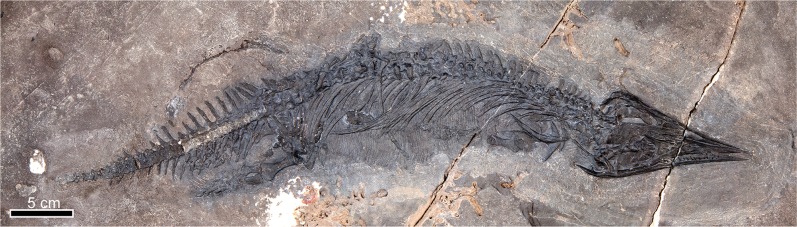
*Gunakadeit joseeae* gen. et sp. nov. Holotype specimen UAMES 23258 in right lateral view.

**Figure 3:**
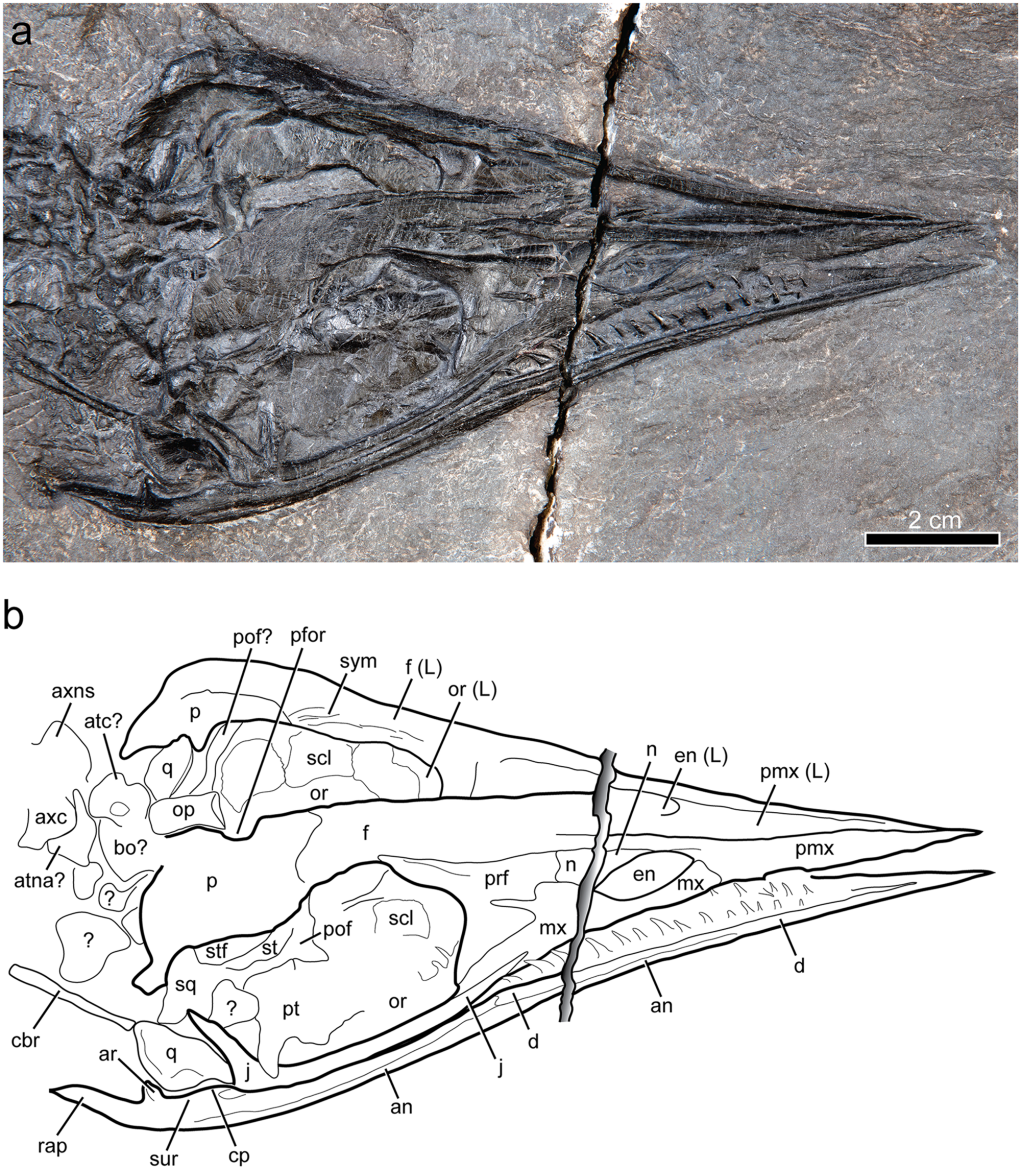
Skull of *Gunakadeit joseeae*. (**a**) Photograph. (**b**) Interpretation of the holotype specimen UAMES 23258 in oblique right lateral view. Abbreviations: an, angular; ar, articular; atc, atlas centrum; atna, atlas neural arch; axc, axis centrum; axns, axis neural spine; bo, basioccipital; cbr, ceratobranchial; cp, coronoid process; d, dentary; en, external naris; f, frontal; j, jugal; L, left; mx, maxilla; n, nasal; op, opisthotic; or, orbit; p, parietal; pfor, pineal foramen; pof, postorbitofrontal; pmx, premaxilla; prf, prefrontal; pt, pterygoid; q, quadrate; rap, retroarticular process; scl, scleral element; sq, squamosal; st, suptratemporal; stf, supratemporal fenestra; sur, surangular; sym, symphysis.

**Figure 4:**
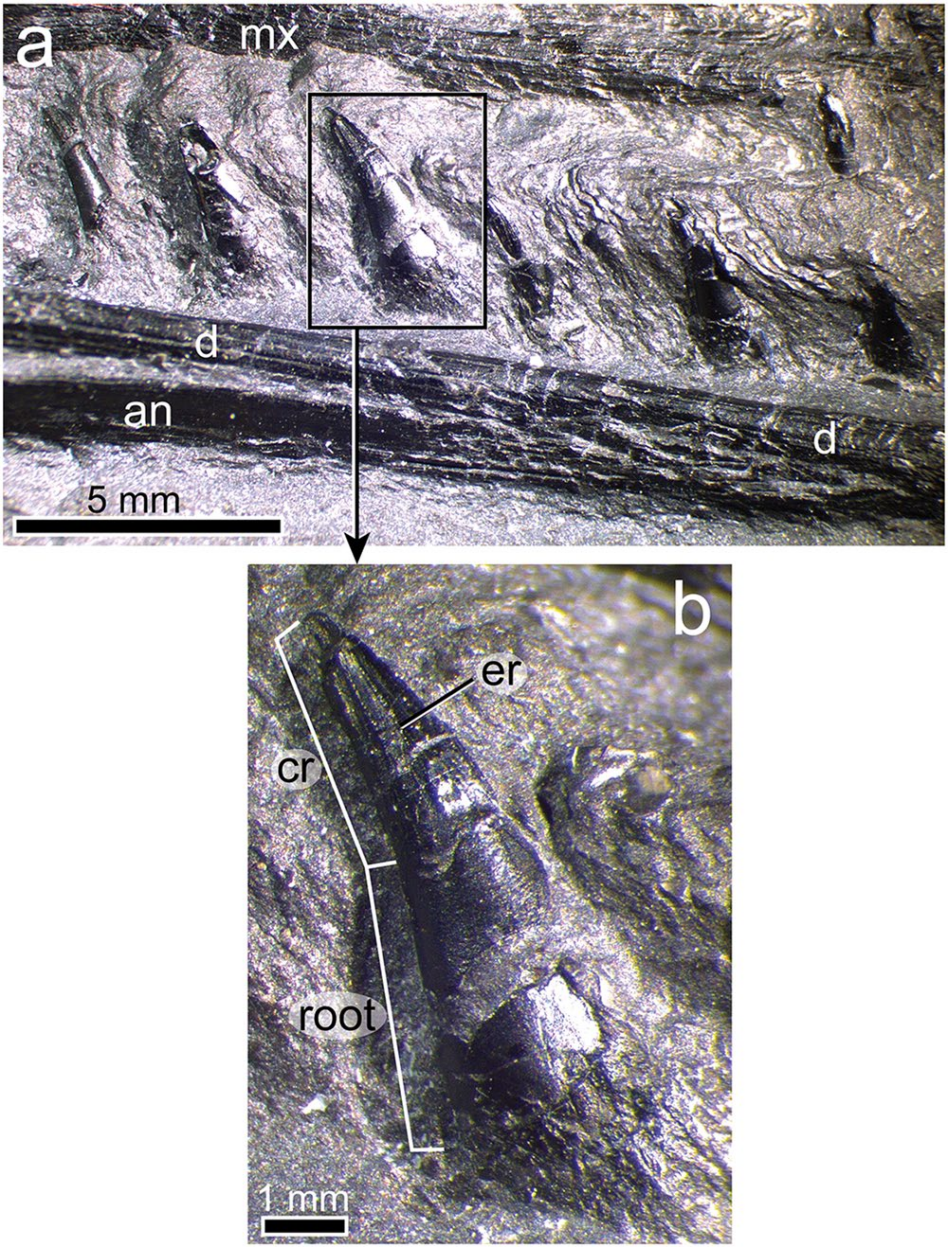
Jaw and teeth of *Gunakadeit joseeae*. (**a**) Photograph of right lower jaw. (**b**) Detail of single dentary tooth of the holotype specimen UAMES 23258 in right lateral view. Abbreviations: an, angular; cr, crown; d, dentary; er, enameled ridge; mx, maxilla.

**Figure 5:**
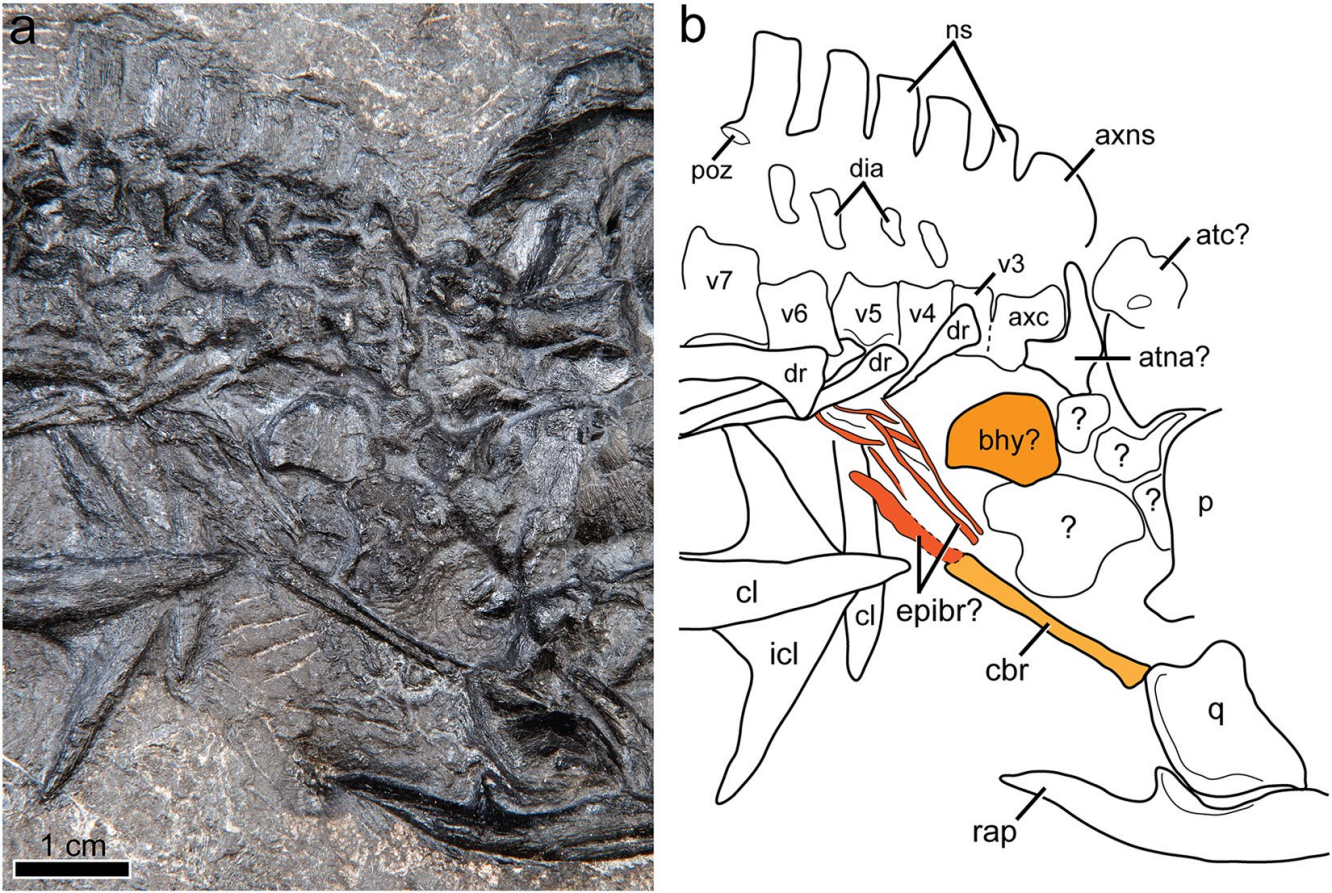
Cervical region and hyoid elements of *Gunakadeit joseeae*. (**a**) Photograph. (**b**) Interpretation of the holotype specimen UAMES 23258 in right lateral view. Colour scheme: light orange, ceratobranchial; dark orange, basihyal; reddish orange, epibranchials. Abbreviations: atc, atlas centrum; atna, atlas neural arch; axc, axis centrum; axns, axis neural spine; bhy, basihyoid; cbr, ceratobranchial; cl, clavicle; dia, diapophysis; dr, dorsal rib; epibr, epibranchial elements; icl, interclavicle; ns, neural spine; p, parietal; q, quadrate; rap, retroarticular process; v, vertebra.

**Figure 6:**
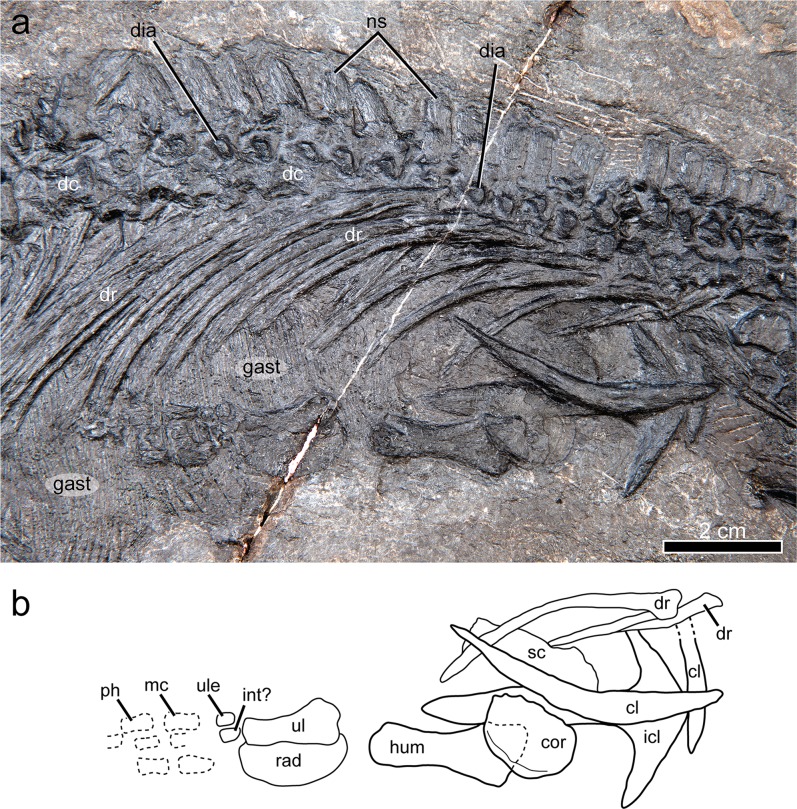
Dorsal region and pectoral girdle of *Gunakadeit joseeae*. (**a**) Photograph. (**b**) Interpretation of the holotype specimen UAMES 23258 in right lateral view. Abbreviations: cl, clavicle; cor, coracoid; dc, dorsal centrum; dia, diapophysis; dr, dorsal rib; gast, gastralia; hum, humerus; int, intermedium; icl, interclavicle; mc, metacarpal; ph, phalanx; ns, neural spine; rad, radius; sc, scapula; ul, ulna; ule, ulnare.

**Figure 7:**
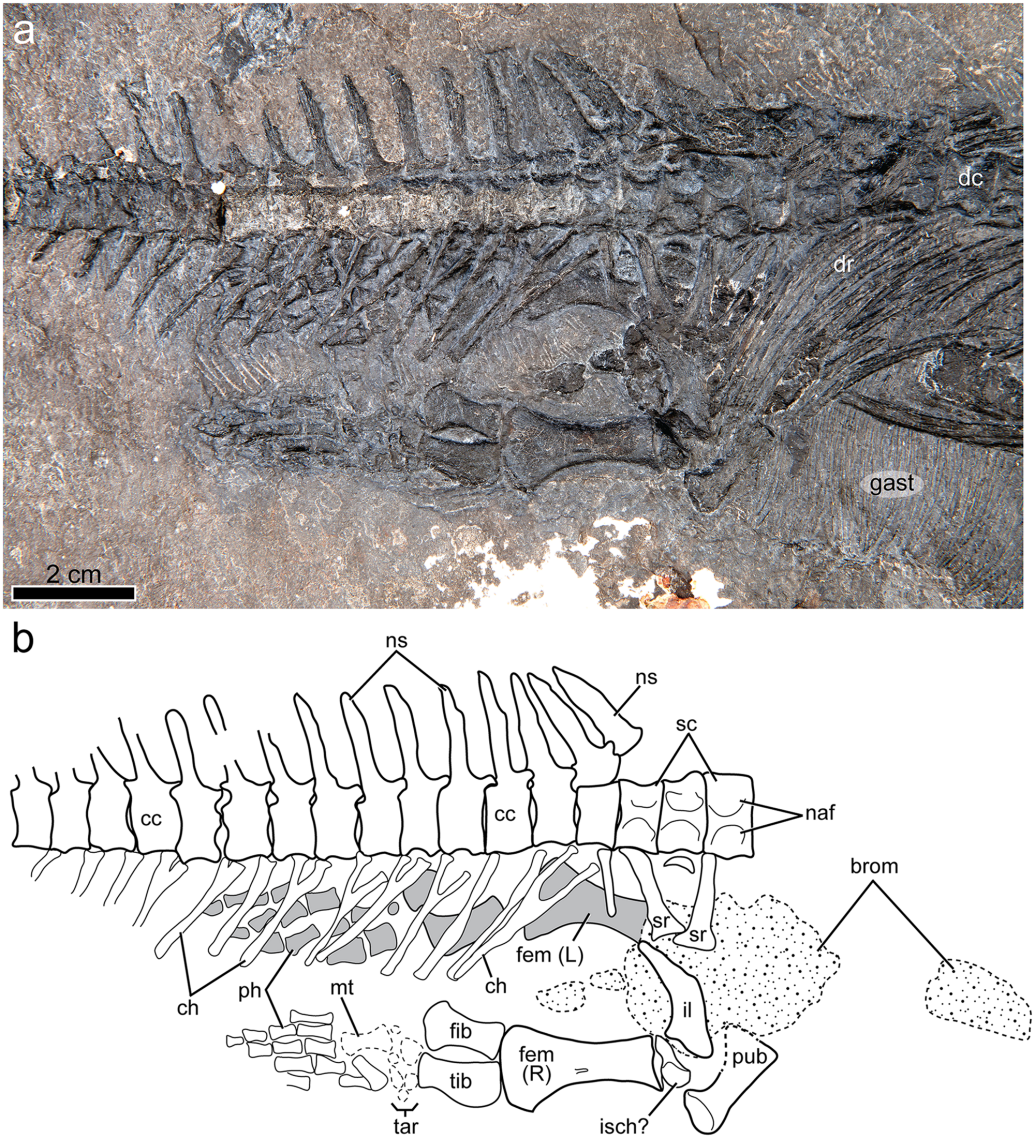
Anterior caudal region and pelvic girdle of *Gunakadeit joseeae*. (**a**) Photograph. (**b**) Interpretation of the holotype specimen UAMES 23258 in right lateral view. Abbreviations: brom, bromalite; cc, caudal centrum; ch, chevron; fem, femur; fib, fibula; gast, gastralia; il, ilium; isch, ischium; L, left; mt, metatarsal; naf, neural arch facet; ns, neural spine; ph, phalanx; pub, pubis; R, right; sc, sacral centrum; sr, sacral rib; tar, tarsal; tib, tibia.

UAMES 23258 (Figs. 2, 3, 4, 5, 6 and 7) a nearly complete skeleton including the skull and most of the axial and appendicular skeleton; missing the distal two-thirds of the tail and the distal portions of the limbs. The holotype is housed in the Earth Sciences Collection at the University of Alaska Museum (UAMES).

### Type locality

Keku Islands of Southeast Alaska (Fig. 1). Precise coordinates are on file with the State of Alaska Office of History and Archaeology.

### Age

*Epigondolella postera* Zone of the Hound Island Volcanics, middle Norian, Upper Triassic.

### Measurements

(Supplementary Tables 1–3).

### Diagnosis

Small-bodied (estimated total length 75–90 cm) thalattosaurian possessing the following autapomorphies: extremely pointed rostrum; posteriorly inclined dorsal process of the jugal; temporal region much shorter than orbit; anterior dentary edentulous; elongate retroarticular process; dorsal, sacral and anterior caudal neural spines with acute dorsal margin; elongate and narrow sacral ribs with distal expansion less than one third rib length; numerous, extremely fine gastralia; tapered posterodorsal process of ilium; tibia preaxial margin strongly convex. *Gunakadeit joseeae* can further be diagnosed on the basis of the following unique character combinations: large external naris; jugal extending far anterior of orbital margin; presence of supratemporal fenestra; reduced cervical region with approximately four cervicals; lateral processes of interclavicle more than 3 times longer than the width of the posterior process; proximal end of humerus wider than distal end; proximodistally elongated reniform radius; posterodistal process of the ilium short; tibia and fibula less than half femoral length.

#### Description and taxonomic comparisons

With the exception of non-fusion along the dorsal midline, the skull remains well articulated with a well-ossified scleral ring, although the visible portions of the braincase are largely disarticulated. Some neural arches have separated from the centra along the neurocentral suture. The carpus and to some extent the tarsus are poorly ossified, although this is conceivably related to their secondarily aquatic lifestyle. These features suggest that the holotype potentially represents a late juvenile or subadult, consistent with its small size (<1 m) compared to most other thalattosaurs.

The skull is triangular in dorsal view with converging lateral margins and lacks any ventral deflection of the rostrum (Figs. 2 and 3). The relative anteroposterior length of the external naris, measured as a percentage of preorbital length, is large in *Gunakadeit* (~20%) compared to typical askeptosauroids (e.g., 8% in *Anshunsaurus huangguoshuensis*^9^) (Supplementary Table 1). The temporal region is much shorter anteroposteriorly than the orbit; in all other thalattosaurians, the temporal region is nearly as long (e.g., *Thalattosaurus alexandrae*^10^) or longer (e.g., *Askeptosaurus*^11^) than the orbit.

The anterior ends of the premaxilla and dentary taper to an acuminate tip, resulting in a straight, narrow rostral morphology most similar to, but more pronounced than, askeptosauroids such as *Askeptosaurus*^11^ and *Endennasaurus*^12^. *Xinpusaurus kohi*^13^ also exhibits an acuminate rostrum, but differs in having a distinct overbite. It is not possible to tell if the anterior end of the premaxilla bears teeth; however, at least one premaxillary tooth is visible anterior to the external naris. The medial surface of the left premaxilla bears a longitudinal groove that extends for nearly its entire length anterior to the external naris, similar to *Xinpusaurus xingyiensis*^14^. The posteromedial process of the premaxilla forms the anterodorsal margin of the external naris and extends posteriorly to the frontals. The posteroventral margin of the premaxilla is overlapped by the maxilla at the anterior margin of the external naris. A shallow trough in the lateral surface of the premaxilla immediately anterior to the external naris results in a shelf that overhangs the anterodorsal margin of the external naris.

The maxilla bounds the entire ventral margin of the external naris, is anteroposteriorly longer than dorsoventrally tall and bears a posterodorsally ascending process overlapping the prefrontal, but not extending to the frontal, as in *Thalattosaurus alexandrae*^10^. A narrow posteroventral process lies along the entire ventral margin of the prefrontal and just contacts the anteroventral margin of the orbit. It is unclear if the maxilla bears teeth. The nasal separates the premaxilla from the maxilla along the posterodorsal margin of the external naris. Its dorsal margin is formed entirely by the premaxilla, thus excluding a contact between the nasals along the dorsal midline, a thalattosaurian synapomorphy^15^. Its anterior end tapers to the dorsal midpoint of the external naris differing markedly from *T. alexandrae*^10^, where the nasal forms the entire dorsal border of the external naris. The posterior end of the nasal abuts the prefrontal; it does not possess the narrow posterior process seen in most thalattosaurians. The prefrontal is robust and forms the entire anterior margin of the orbit. Ventrally, the prefrontal approaches, but does not contact the jugal, as in *Clarazia*^16^. A distinct, anteroposteriorly elongate ridge on its dorsolateral surface produces a prominent shelf of bone extending over the anterodorsal margin of the orbit. The lacrimal is absent. The gracile jugal bounds the entire ventral and posteroventral margins of the orbit. Its anterior end extends anterior to the orbit, similar to *Clarazia*^16^ but in contrast to *Askeptosaurus*^11^ and *Miodentosaurus*^6^ that terminate in line with, or posterior to the orbital margin, respectively. The dorsal process of the jugal is inclined posteriorly, not vertically, as in most thalattosaurians. The posterior process of the jugal is very reduced or possibly absent given that the dorsal process abuts the anterior margin of the quadrate similar to *Xinpusaurus xingyiensis*^14^ but in marked contrast to most askeptosauroids.

The poorly preserved postorbitofrontal appears to be a single ossification. It has a very short descending process to meet the very tall ascending process of the jugal, although it may be shortened due to breakage. The postorbitofrontal seemingly lacks an extensive anterior process, or it may be covered by the parietal. The anterior end of the frontal appears to be split by a posterodorsal process of the premaxilla, although poor preservation makes their exact relationships hard to discern. The frontal forms most of the dorsal rim of the orbit and bears a short, broad posterolateral process that may barely contact the postorbitofrontal. Along the dorsal midline, the medial surface of the frontal is visible and is surprisingly thick dorsoventrally (3–4 mm), bearing narrow, posteroventrally-sloping ridges along the symphysis, just anterior to the frontal-parietal suture. The frontal-parietal contact is sigmoidal. The parietal overlaps the frontal and encloses a prominent pineal foramen approximately in line with the postorbital bar. The anterolateral border of the parietal is formed by the supratemporal and postorbitofrontal and may contribute to the orbital border. A distinct posterolateral process is visible and appears to contact a distinct element, likely the squamosal, posterolaterally.

The anteroposteriorly elongated supratemporal is medial to, and overlaps the postorbitofrontal. The supratemporal extends anteriorly almost to the posterior margin of the frontal, possibly contacting it as in *Clarazia*^17^. The supratemporal forms the lateral border of a small but distinct opening interpreted to be the supratemporal fenestra, which is bordered medially by the parietal. The supratemporal fenestra is approximately 6 mm long and 2 mm wide, but not slit-like as interpreted for *Thalattosaurus alexandrae*^10^. Its position differs from askeptosauroids (e.g., *Askeptosaurus*) where it is bounded laterally by the postorbital and medially by the parietal, and other thalattosauroids (e.g., *Thalattosaurus*), where it is bounded laterally by the postorbitofrontal and medially by the frontal and supratemporal. Alternatively, the lateral border of the supratemporal fenestra may be formed entirely by the postorbitofrontal, which is difficult to distinguish from the supratemporal due to preservation. The quadrate abuts the posterior end of the jugal, exemplifying the extremely shortened temporal region. As preserved, the lateral surface is smooth, lacking the obvious ridges observed in *Xinpusaurus suni*^18^. The posterior margin is convex; however, much of its morphology is difficult to discern. Several small and disarticulated bones in the posterior skull region may be braincase elements. One of the larger elements near the atlas-axis complex may represent the basioccipital and another the left opisthotic. Exoccipital, prootic, supraoccipital and stapes were not identified.

A portion of the palate is visible through the right orbit. The lateral ramus of the pterygoid lies in proximity to the base of the dorsal process of the jugal. The exposed dorsal surface of the pterygoid is otherwise largely flat. The presence of palatal dentition (pterygoid or palatine) is unknown. Portions of both the left and right scleral rings are visible; the left ring includes at least three articulated, subrectangular scleral ossicles. Each ossicle thins toward the outer part of the ring and bears a radially striated surface texture. Based on ossicle proportions and the apparent diameter of the ring, there were likely fewer than the estimated fourteen scleral ossicles in *Askeptosaurus*^11^ or fifteen estimated in *Concavispina*^19^.

The left and right dentaries are fused along the midline symphysis for at least one third of their entire length, similar to *Endennasaurus*^12^ but unlike the shorter and unfused symphysis of *Clarazia*^17^. The anterior one-third of the dentary is clearly edentulous, an autapomorphy of this taxon. The dentary toothrow is straight. It contacts the surangular at a butt joint immediately posterior to the last visible tooth. The dentary-angular (or possibly the dentary-splenial) suture may occur approximately halfway along the length of the dentary. If this is the actual suture, the angular extends farther anteriorly than in most thalattosaurs, with the possible exception of *Concavispina*^19^. Posteriorly, the angular forms the ventral margin of the mandibular ramus at least as far as the mandibular fossa. The angular may also form some or all of the retroarticular process, although a distinct angular-articular suture is not visible. The retroarticular process is very elongate and dorsomedially deflected to a degree not seen in any other thalattosaur. The posterior margin of the mandibular fossa is marked by a mediolateral ridge; posterior to this the dorsal surface of the retroarticular process is relatively flat. The surangular forms the dorsal margin of the mandibular ramus and is bordered ventrally by the angular for its entire length. The coronoid process is a low mediolaterally thickened eminence, differing from the distinct and dorsally elongate process of *Clarazia*^16^. A distinct element identifiable as the coronoid is absent. A small, trough-like depression is located near the apex of the coronoid process. The posterior margin of the angular is marked by a suture visible just ventral to the glenoid fossa.

Fourteen teeth are visible in the right dentary and five in the left (Fig. 4). The dentition is homodont and each tooth consists of a slender, conical and recurved crown that is round in cross section. Tooth height increases posteriorly and the largest crowns are distributed throughout the posterior half of the toothrow, with the largest tooth being dentary tooth 13. Emergent replacement teeth are not visible. In the well-preserved eighth right dentary tooth, the crown comprises one-third of total tooth height and bears 4–5 straight enameled ridges from the base of the crow to the apex on the labial surface. A slight flattening on the apical surface of the crown may represent faint tooth wear. The surface of the root is smooth and expands in circumference basally. Roots are slightly mediolaterally flattened, presumably due to crushing of the pulp cavity. There is no evidence of distinct alveoli or a bony septum between adjoining teeth. Rather, the teeth appear to be set deeply against the medial surface of the jaw and thus tooth implantation is likely best described as pleurodont, as suggested for *Clarazia*^16^. It cannot be determined whether any type of shelf or lingual wall supports the roots, but some degree of bony attachment of teeth seems likely given that teeth show little sign of displacement.

A well-developed hyoid apparatus includes a prominent rod-like element, interpreted as ceratobranchial 1, and at least three additional thinner, rod-like elements preserved in close association just anterior to the clavicular arch (Fig. 5). Ceratobranchial 1 is minimally 18 mm in length (partially covered by the quadrate), straight, and slightly larger in circumference at its posterior end. Immediately posterior to it is another poorly ossified element, possibly epibranchial 1, which is 10 mm in length. Given its poor preservation it may have been only partly ossified, or even cartilaginous in life, as is typical of many modern amniotes^20^. At least two other poorly ossified and thin rod-like structures lie adjacent and parallel to epibranchial 1, possibly representing portions of neighboring hyoid arches. Lying in close proximity to these hyoid elements is an isolated plate-like bone that may represent the ossified corpus of the hyoid. Consistent with its identity is its overall shape, although it is not perfectly bilaterally symmetrical, and that it is apparently unpaired and lies in an appropriate relative position to other elements. In other thalattosaurians for which a hyoid apparatus is known (e.g., *Anshunsaurus huangguoshuensis*^21^; *Concavispina*^19^) only a single pair of elongate elements (i.e., certatobranchial 1) have been described.

The articulated axial column comprises 58 vertebrae, including a complete series of 32 presacral and three sacral vertebrae (Figs. 6 and 7). The incomplete tail preserves 23 partial vertebrae. Four cervical vertebrae, including axis and atlas, are identified; cervicals 3 and 4 are recognized on the basis of possessing short and straight ribs that are not long enough to have contacted the unossified sternum. The neck of *Gunakadeit* is very short compared to askeptosauroids such as *Anshunsaurus wushaensis* (15–16 cervicals^22^) or even short-necked thalattosauroids such as *Hescheleria* (7–8 cervicals^23^), but is comparable to *Concavispina* (~4 cervicals^19^). The atlas-axis complex is not fully articulated. The axis is identified by its anteroposteriorly expanded neural arch and attendant centrum. The atlas is represented by at least two elements lying immediately anterior to the axis and posterior to the skull; the larger element is possibly the atlantal centrum and a smaller, wedge-shaped atlantal neural arch. The identities of other smaller elements in this vicinity are uncertain. In lateral view, the axial centrum and its neural spine are anteroposteriorly longer than dorsoventrally tall. Atlantal and axial ribs were not identified, but the axis bears a prominent single-headed diapophysis on its ventrolateral surface.

The dorsal centra increase slightly in length in the anterior half of the dorsal series (from approximately 5 mm to 6–7 mm), and then remain relatively uniform in length into the sacrals. The ventral portion of the dorsal neural arches is robust and bears a single large, anteroventrally to posterodorsally elongate rib facet. The cervical and anterior dorsal neural spines are vertically oriented and evenly rectangular in outline, being approximately twice as tall as long, similar to *Xinpusaurus suni*^24^. However, throughout the posterior half of the dorsal series the neural spines become angled posteriorly and the apices of the neural spines are conspicuously pointed in lateral view, an autapomorphy of *Gunakadeit*. This contrasts with the dorsally-notched spines of *Concavispina*^19^, the short and broad spines of *Askeptosaurus*^11^ and the strongly constricted ventral portion of the neural spines in *Anshunsaurus huangguoshuensis*^9^. All of the visible rib heads are single-headed. The anterior-most rib associated with vertebra 3 is very short, being approximately two times the length of the centrum. On vertebra 4, the rib becomes longer (~20 mm) but remains relatively straight. The more posterior dorsal ribs are considerably longer (up to ~70 mm total length) and robust, are round in cross-section at midshaft, and distally flattened.

Three sacral vertebrae are distinguished by the presence of articulated sacral ribs on the first and third centra. Three sacrals are also found in *Miodentosaurus*^25^ and *Concavispina*^19^ while only two are present in *Endennasaurus*^12^ and *Xinpusaurus* sp.^13^. The sacral centra have similar proportions to the posteriormost dorsals, unlike *Miodentosaurus* where the sacrals are somewhat longer^25^. The sacral ribs are long, straight and possess a narrow, rounded shaft proximally. Distally, the ribs become dorsoventrally flattened and anteroposteriorly expanded. The width of the distal expansion is less than one third the entire length of the rib resulting in ribs that are relatively elongate, even compared to *Endennasaurus*^12^. The absence of a sacral rib on the second centrum may reflect primary absence or taphonomic loss.

Caudal centra are only slightly shorter than sacrals and posterior dorsals. A caudal rib may be associated with the first caudal centrum. The neural spines are narrowly rectangular in lateral view and display a slight anteroposterior expansion in the dorsal half of the spine. The spines are much taller than the height of the centrum, but not as comparatively tall as those in the Kössen Formation specimen (SMNS 90568) from Austria^26^. The lateral surfaces of the spines bear faint striations but lack the more prominent grooves seen in SMNS 90568^26^. The proximal spines are nearly vertically inclined but become posteriorly inclined and shorter, starting around caudals 6 and 7. The spine apex is not as pointed as those in the dorsal series. Chevrons on the first caudal vertebra are very long, more than two times the height of the centrum, but again not as comparatively long as those in SMNS 90568^26^, and diminish in length posteriorly.

The entire articulated gastral basket is preserved with the first gastralia appearing immediately posterior to the posterior end of the interclavicle and extending through the remainder of the torso, terminating just anterior to the pelvic girdle. Individual gastralia are extremely delicate and almost hair-like in appearance, making them difficult to precisely count. The gastralia are more gracile that those known from any other thalattosaurian, including the Kössen Formation specimen and *Concavispina*, which has approximately 100 pairs^19^. There appears to be a set of lateral elements, at least 70 in number, that overlap with a set of medial elements. Where visible, the lateral and medial elements are gently curved to nearly straight in shape. Two homogenous phosphatic masses are located adjacent to the pelvic girdle. Although lacking identifiable contents, their composition, shape and position suggest they are bromalites (gastric residues).

The specimen preserves both clavicles and coracoids, the interclavicle, and a partial right scapula (Fig. 6). The interclavicle is robust and T-shaped; however, the anteriormost tip is covered and it is unknown whether it possesses an anterior process, as in *Clarazia*^17^. The entire anterior margin is convex and the lateral processes are angled posteriorly at ~30 degrees. The lateral processes are conspicuously elongate; the length of each process is more than three times longer than the width of the mid portion of the posterior process, in contrast to most thalattosaurians (e.g., *Endennasaurus*^12^). The posterior process is mediolaterally widest at mid-length. The right clavicle is very long, being as long as the interclavicle and twice as long as the humerus, similar to *Concavispina*^19^, and is markedly robust midshaft, unlike *Anshunsaurus huangguoshuensis*^21^. The dorsal portion of the clavicle loosely contacts the scapula and terminates slightly beyond its posterior margin. As preserved, the scapula is anteroposteriorly longer than broad. The right coracoid is oval in shape, slightly anteroposteriorly longer than broad and is less than half as long as the interclavicle. The element is generally thin but appears slightly thickened along its posterolateral margin, in the vicinity of the glenoid. On its surface are a series of radiating, concentric lines. A coracoid foramen could not be identified.

The proximal end of the right humerus is wider than the distal end in contrast to askeptosauroids such as *Miodentosaurus*^27^. There appears to be no torsion in the shaft in contrast to *Nectosaurus*^10^, where the distal end of the humerus is twisted relative to the proximal end. A moderately developed deltopectoral crest may be present; however, it does not resemble the thin, flange-like crest seen in *Thalattosaurus alexandrae*^10^. Distally, the radial facet appears better developed than the ulnar facet and a shallow trough separates the two condyles. The well-developed radius and ulna are approximately 0.7 times the proximodistal length of the humerus. The radius is reniform, with a prominent convex preaxial margin, unlike the elongate radii seen in askeptosauroids. The similarly reniform radius of *Xinpusaurus xingyiensis*^14^ is proportionately shorter, being ~0.5 times the length of the humerus. The radius also lacks a distinct notch near its proximal end, as seen in *Nectosaurus*^10^. The proximal end of the ulna is much wider than the distal end, similar to *Clarazia*^17^ but it lacks a distinct olecranon process, in contrast to *Nectosaurus*^10^. The pre- and postaxial margins of the ulna are concave. Two proximal elements of the carpus are ossified; based on their position they may be the ulnare and intermedium. There appears to be a minimum of four digits present, but the rest of the autopodium is poorly preserved.

The ilium is curved in lateral view, with a well formed but short posterodorsal process that is less than two sacral vertebrae in combined length (Fig. 7). The dorsal margin is straight and lies in close proximity to the distal ends of the sacral ribs, but the ventral margin is gently curved, resulting in a tapering, wedge-shaped posterodorsal process, an autapomorphy of the taxon. In contrast, askeptosauroids such as *Endennasaurus*^12^ have nearly parallel-sided dorsal and ventral margins that only taper at the posteriormost end. In thalattosauroids such as *Concavispina*^19^ the posterior end of the posterodorsal process is slightly expanded in dorsoventral height and is also relatively longer, being more than twice the combined length of two sacrals. The acetabular end is the widest portion of the element. The acetabular surface is partly concealed but the lateral margin of the acetabulum is concave with a narrow, tapering anterior margin for the pubis and a ventrally deflected margin at the ischial end. What is tentatively identified as the pubis is situated ventral and anterior to the ilium and slightly overlaps the posteriormost gastralia. The acetabular end is robust and is set off from the thinner and anteroposteriorly expanded medial portion by a distinct shaft, which has a straight anterior margin and a concave posterior margin. An obturator foramen is absent. In contrast, the pubis of other thalattosaurians is typically mediolaterally shorter and more plate-like in overall appearance (e.g., *Hescheleria*^17^) and bears a distinct obturator foramen. Given the aberrant morphology of this element, it might alternatively represent a displaced sacral rib or another element. The ischium is largely not visible.

The femur is nearly identical in length to the humerus (Supplementary Tables 2 and 3) and paddle-shaped, with the distal end being conspicuously wider than the proximal end, similar to *Thalattosaurus alexandrae*^10^. The femur lacks any obvious proximal trochanters (internal or fourth), which are prominent in *Miodentosaurus*^25^ and *Hescheleria*^17^. The shaft is distinctly constricted and there is a midshaft depression that might be for insertion of the adductor musculature. The distal articular surface is evenly convex lacking distinct articular facets for the tibia or fibula. The tibia and fibula are about equal in length but less than half the length of the femur; in contrast, the tibia and fibula are approximately half as long as the femur in *Clarazia*^17^ or more than half as long in *Anshunsaurus huangguoshuensis*^21^. The tibia possesses an autapomorphic convex preaxial margin. The fibula is broadly expanded midshaft but both pre- and postaxial margins are convex. Its distal end is only slightly more expanded than its proximal end; in contrast, the distal end of the fibula is markedly expanded in *Clarazia*^17^. Six or seven elements are visible in the tarsus, but are poorly preserved and/or ossified. The largest is the astragalus, which lies distal to the spatium osseum and in contact with both the tibia and fibula. A small unidentified element is located preaxially of the astragalus; distal to these proximal tarsals are three (possibly four) distal tarsals. A small ossicle located posterodistal to the astragalus may be the calcaneum. Only two probable metatarsals (I and II?) are present and there are 12 proximal phalanges still in articulation, indicating five digits in the hind limb.

### Phylogenetic position

**Figure 8:**
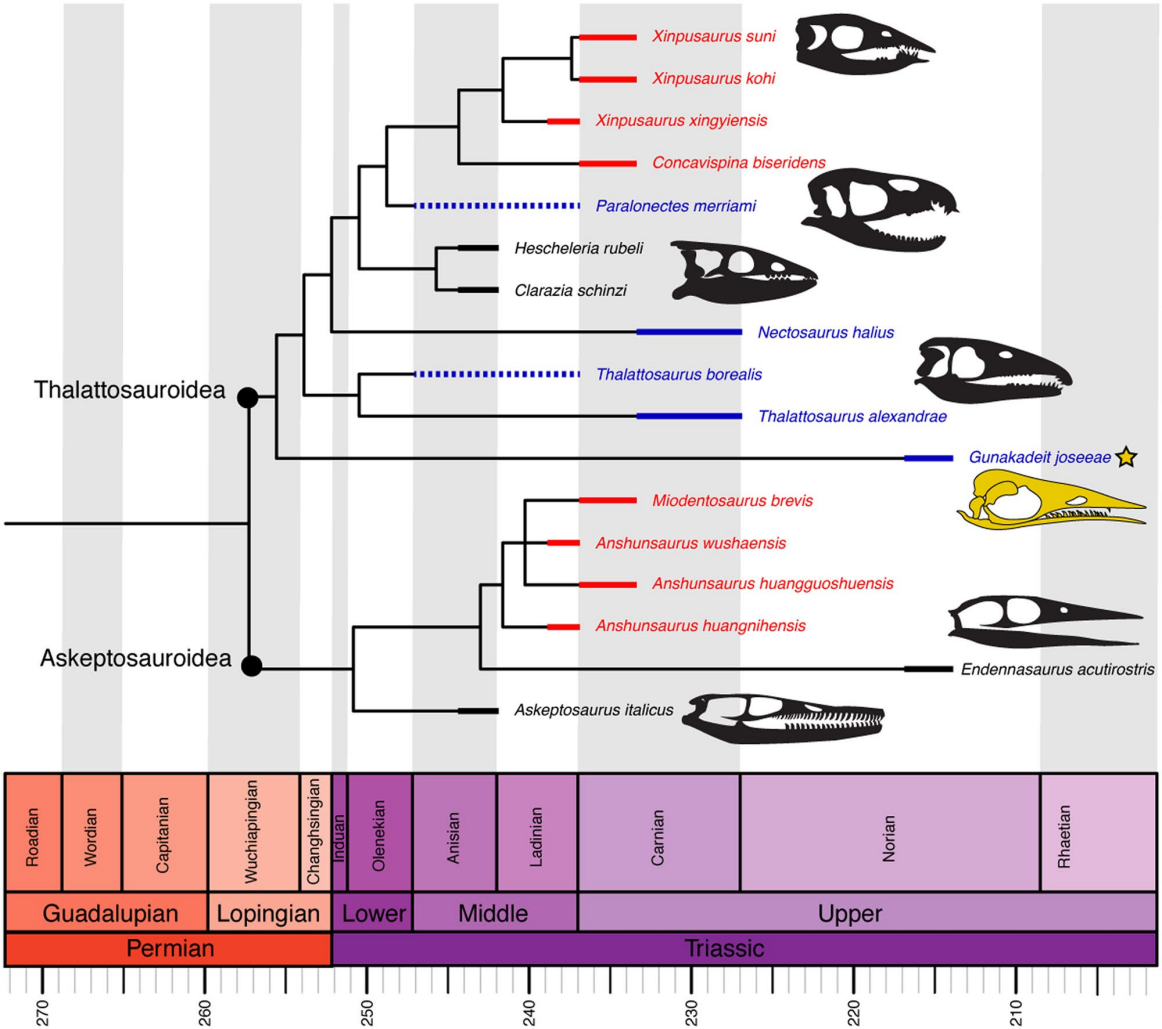
Time-scaled strict consensus topology of Thalattosauria. Thickened lines represent age range of each taxon; dashed line represents range of uncertainty. Colour scheme: red, Eastern Tethys (modern China); blue, Western Tethys (modern Europe); black, eastern Panthalassa (modern western North America).

After the deletion of three highly incomplete taxa (see Methods) our strict consensus tree (Fig. 8) is nearly completely resolved, with the exception of a polytomy among *Anshunsaurus huanggoushuensis*, *A*. *wushaensis* and *Miodentosaurus*. A monophyletic Thalattosauria is recovered with a basal dichotomy splitting all ingroup OTUs into one of two traditionally recognized clades, Askeptosauroidea and Thalattosauroidea, in accordance with most previous analyses. *Gunakadeit joseeae* is recovered as the basalmost member of Thalattosauroidea. Bootstrap values indicate strong support for the monophyly of Thalattosauria (99%) and Thalattosauroidea (88%); however, support for internal branches is generally less than 50%, with the exception of the sister group relationship between *Thalattosaurus alexandrae* and *T*. *borealis* and *Xinpusaurus kohi* and *X*. *suni* (both 58%).

## Discussion

*Gunakadeit* expands the already striking morphological disparity seen in thalattosaurs, indicative of adaptation to a wide range of dietary and ecological roles^4^. In our phylogenetic analysis, *Gunakadeit* is recovered as the earliest diverging thalattosauroid, seemingly at odds with its stratigraphic occurrence as one of the youngest thalattosauroids and one of the last known thalattosaurs worldwide (Fig. 8). This implies a ≥ 20 million-year ghost lineage between *Gunakadeit* and the earliest known thalattosauroids, either the latest Anisian-aged *Hescheleria* and *Clarazia*^17^ or the poorly age-constrained *Thalattosaurus borealis* and *Paralonectes* (Spathian? or middle Triassic)^28^.

Despite this stratigraphic incongruence, *Gunakadeit* exhibits a mosaic of features consistent with its relatively basal position among thalattosauroids. *Gunakadeit* shares several synapomorphies with other thalattosauroids, particularly in the postcranium, including tall anterior caudal neural spines, a distally expanded femur and kidney-shaped radius. In contrast, many cranial characteristics, including the homodont dentition, straight alveolar margin, tapered rostrum lacking ventral deflection, and short coronoid eminence more closely resemble askeptosauroid features. While these similarities with askeptosauroids could be homoplastic, perhaps reflecting a similar feeding ecology, several are also observed in outgroup taxa indicating that *Gunakadeit* may retain some cranial plesiomorphies lost in other thalattosauroid lineages. Biogeographically, it is interesting that the more aquatically adapted thalattosauroids were globally distributed whereas the more plesiomorphic askeptosauroids were apparently restricted to the Tethyan realm (Fig. 8). *Gunakadeit’s* phylogenetic position indicates that trans-hemispheric distribution occurred early in thalattosauroid evolution, and dispersals events across Panthalassa may have happened more than once. Additionally, *Gunakadeit* shows that postcranial aquatic specializations among thalattosauroids (e.g., shortened limbs, elongate neural spines) likely preceded cranial specializations (e.g., heterodonty, rostral deflection) that typify more derived thalattosauroids.

Based on tooth morphology and the dietary classification proposed by Massare^29^, *Gunakadeit* falls into the ‘Pierce II’ guild (relative tooth size ~0.06, tooth shape index = 2.0) consistent with a diet of soft cephalopods or small fish. Unfortunately, the preserved gut contents provide no identifiable prey remnants; however, the absence of shell, bone or scales is consistent with a soft-bodied prey preference. The well-developed and ossified hyoid apparatus, including an ossified plate-like basihyal, as well as an elongate retroarticular process suggestive of rapid jaw depression, could indicate an adaptation toward suction generation^30^ or to support lingual musculature involved in chemosensation or food-transport^31^. *Gunakadeit* might have ambushed small soft-bodied prey within the water column and/or probed for small prey in cavities and crevices, capturing prey via suction or prehension using acute, forceps-like jaws. Soft prey would be grasped with the pointed, recurved teeth prior to being swallowed whole with assistance from some combination of suction, a muscular tongue and/or inertial feeding. This scenario is consistent with the paleoenvironmental setting in a volcanic island arc fringed with coral reefs (see Methods).

*Endennasaurus*, another late surviving thalattosaur from the Norian of Italy, occupies a similarly deeply nested position among askeptosauroids (Fig. 8). Both were relatively small forms with tapering skulls that likely targeted small, soft-bodied prey^12^. However, *Endennasaurus* exhibits a relatively plesiomorphic postcranium compared to *Gunakadeit*. The poorly ossified carpus and tarsus, flattened zeugopodia and exceptionally elevated caudal neural spines in *Gunakadeit* reflect increased dedication to aquatic life and would have made terrestrial locomotion awkward, whereas *Endennasaurus* retained well-formed limbs and may have been capable of occasional terrestrial locomotion^12^.

*Gunakadeit* exemplifies the varied specializations thalattosaurs evolved that allowed them to proliferate across coastal environments during the Triassic. Despite their broad dispersal and ecomorphological variety, thalattosaurs failed to adapt to the highly pelagic lifestyles observed in contemporaneous Late Triassic parvipelvian ichthyosaurs^32^ and plesiosaurian sauropterygians^33^ that persisted into the Jurassic. This apparent selectivity of marine reptile extinction across the Triassic-Jurassic boundary may be tied to changes in sea level and/or reorganization of marine ecosystems during the end-Triassic mass extinction^3,34^. Lagerstätten effects and poor sampling, especially in the Norian, likely exert a strong influence on the thalattosaurian fossil record, biasing a more complete understanding of their evolutionary history^1,34^. Nevertheless, the persistence of two superficially similar but phylogenetically and geographically disparate forms into the Norian may point to declining diversity and a relatively limited ecological breadth among Late Triassic thalattosaurs, culminating in their eventual extinction by the end of the Triassic.

## Methods

### Specimen and preparation

UAMES 23258 was found in an intertidal outcrop in the Keku Strait area of southeast Alaska (Fig. 1). It is fully articulated and nearly complete (Fig. 2), with the exception of posterior two-thirds of the tail, which was lost due to erosion. The carcass came to rest on the seafloor largely on its ventral/left side, and was prepared on the surface stratigraphically up, providing a mostly unobstructed view of the right side of the skeleton. The skeleton was mechanically prepared using a combination of air scribes and air abrasives.

### Geological context

Much of southeastern Alaska consists of allochthonous terranes that accreted to the continental margin during the Mesozoic and Cenozoic^35–39^. The Alexander Terrane is one of the largest of these displaced tectonic fragments and includes several units deposited within a volcanic island-arc complex^40^. UAMES 23258 was preserved in a sedimentary package comprising part of the Hound Island Volcanics (HIV). The HIV is part of the Upper Triassic Hyd Group and consists of basaltic pillow lava and pillow breccia, massive basalt, hyaloclastic tuff, limestone and volcaniclastic sandstone^41,42^. The HIV conformably overlies the shallow water carbonates of the Cornwallis and Hamilton Island Limestones. Paleomagnetic data suggest the Alexander terrane was located at approximately 10–20 degrees north latitude during deposition of the HIV in the Late Triassic^43,44^.

The outcrop from which the specimen was found consists of alternating units of volcaniclastic-rich bioclastic limestone and calcareous shale. The bioclastic limestones (Facies 1 of Adams^45^) contain a diverse assortment of disarticulated marine vertebrate remains, including small thalattosaur elements. UAMES 23258 was found in a *Halobia*-rich calcareous shale (Facies 2 of Adams^45^). The shale was deposited in protected waters which periodically received debris flows from an adjacent shelf, all proximal to active volcanism. Just a few meters from UAMES 23258 is a volcanic bomb that impacted the calcareous muds distorting the surrounding layering. The high degree of skeletal articulation suggests little to no post-mortem transport of the specimen.

The age of the HIV is well constrained on the basis of biostratigraphic relationships^41^. Samples from the study site contain the bivalve *Halobia fallax* and the conodonts *Epigondolella postera*, and *E. spiculata*^45^. Collectively, these taxa constrain the new thalattosaur specimen within the *Epigondolella postera* Zone, indicating a middle Norian age^42,46–48^.

### Phylogenetic analysis

For this study, we constructed a completely new phylogenetic matrix consisting of 78 characters and 23 OTUs (Operational Taxonomic Units; three outgroup and 20 ingroup; Supplementary Note 2). Our matrix builds on, and nearly doubles the number of characters used in the most recent analysis of thalattosaurian relationships by Li *et al*.^14^, which in turn is based on the data matrices of Nicholls^10^, Liu and Rieppel^21^, Müller^26^ and Liu *et al*.^19^. All previously used characters were critically reevaluated; some were either reworded and/or redefined for clarity and in other instances character states were modified, added or deleted. Additionally, a total of 37 new characters were developed in the building of this matrix (23 cranial, 14 postcranial). The newly created phylogenetic character list (Supplementary Note 2) attempts to capture morphological variation mentioned in other descriptive works but that had not been previously incorporated into discrete phylogenetic characters, as well as anatomical insights gained in the study of the new Alaskan taxon.

With one exception, our matrix includes all ingroup OTUs used in the analysis of Li *et al*.^14^; pending a more complete revision of the species-level taxonomy of *Xinpusaurus*, only three of the four named species in this genus were selected, excluding *X. bamaolinensis*. To this list we added three new OTUs including the nearly complete Alaskan specimen, UAMES 23258, and two unnamed, postcranial specimens; TMP 88.99.21 from the Sulphur Mountain Formation of British Columbia, Canada^28^ and SMNS 90568 from the Kössen Formation of Austria^26^. Fifteen of the 20 ingroup taxa were scored based on personal inspection and scores for other taxa were based on the literature, augmented by photographs and notes from colleagues. Whenever possible, the holotype specimen served as the primary data source, but in some instances formally referred material was also used. Given the uncertainty regarding placement of Thalattosauria within Diapisda, three basal diapsids, *Petrolacosaurus kansensis* (Carboniferous), *Youngina capensis* (Permian) and *Claudiosaurus germaini* (Permian) were selected as outgroups.

Parsimony analyses were performed in PAUP* 4.0 (build 165) for Macintosh^49^. *Petrolacosaurus*, *Youngina* and *Claudiosaurus* were defined as outgroups. For each heuristic search starting trees were acquired by stepwise addition with random addition sequence and TBR branch swapping. Branch support was calculated using bootstrap percentages in PAUP*. Analysis of the full dataset (23 taxa, 78 characters) results in a strict consensus tree (of 44 trees at 192 steps) with a large polytomy among thalattosauroids (Supplementary Note 2). To test the effect of missing data, three highly incomplete OTUs (*Agkistrognathus*, TMP 88.99.21 and SMNS 90568) were pruned and the analysis repeated. The complete matrix is provided in Supplementary Data 1.

### Nomenclatural acts

This published work and the nomenclatural acts it contains have been registered in ZooBank, the proposed online registration system for the International Code of Zoological Nomenclature. The ZooBank LSIDs (Life Science Identifiers) can be resolved and the associated information viewed through any standard web browser by appending the LSID to the prefix “http://zoobank.org/”. The LSIDs for this publication are: urn:lsid:zoobank.org:pub:4B8E7305-F27E-4DC5–8987–6AC6CBDD3C75; urn:lsid:zoobank.org:pub:70873F20-1D78-4929-9FDA-7C4855269224; urn:lsid:zoobank.org:pub:F0CDE318-14ED-458A-B935-D1C0E7890AF3.

## Supplementary information

Supplementary Information.

Supplementary Dataset 1.
