# Supplementary material for: An articulated Late Triassic (Norian) thalattosauroid from Alaska and ecomorphology and extinction of Thalattosauria
Source: Sci Rep. 2020 Feb 4;10:1746. doi: 10.1038/s41598-020-57939-2 (PMC7000825; doi:10.1038/s41598-020-57939-2)
Supplement: Supplementary file 1 — Supplementary Information. [file 41598_2020_57939_MOESM1_ESM.pdf]

Supplementary Information

An articulated Late Triassic (Norian) thalattosauroid from Alaska and ecomorphology and extinction of Thalattosauria

Patrick S. Druckenmiller, Neil P. Kelley, Eric T. Metz and James Baichtal

Contents

Supplementary Tables

1. Cranial measurements……………………………………………………………………………3
2. Pectoral girdle and forelimb measurements…………………………….…………………3
3. Pelvic girdle and hindlimb measurements……………………………………………………4

Supplementary Note 1: Institutional Abbreviations………………………………………………………5

Supplementary Note 2: Phylogenetic Analysis

1. Character List……………………………………………………………………………………….5
2. Taxon-character data matrix scores……………………………………………………...15
3. Analysis and results……………………………………………………………………………….17

Supplementary References…………………………………………..…………………………………..…19

Supplementary Table 1. Selected cranial measurements of UAMES 23258, holotype specimen of *Gunakadeit joseeae* (in mm). Abbreviations: L, left; R, right.

Total skull length (dorsal midline)	109.3
Maximum skull width (R x 2)	42.2
Preorbital length	69.9
External naris length	13.1
Orbit length (L)	31.1
Pineal foramen width (R x 2)	5.0
Mandibular ramus length (R)	131.9
Mandibular symphysis length	24.2

Supplementary Table 2. Selected measurements of the pectoral girdle and forelimb skeleton of UAMES 23258, holotype specimen of *Gunakadeit joseeae* (in mm). Measurements of paired elements taken from right side. *denotes estimated value.

Clavicle length	46.0
Clavicle width	5.0
Interclavicle mediolateral width	30.5
Interclavicle anteroposterior length	45.0
Coracoid length	17.5
Coracoid width	12.0
Scapula minimum length*	23
Forelimb length*	70.5
Manus length*	26.5
Manus width at metacarpals*	10.5
Humerus anteroposterior length	26.0
Humerus maximum proximal width	11.0

Humerus maximum distal width	7.0
Radius length	16.5
Radius width	7.5
Ulna length	16.5

Supplementary Table 3. Selected measurements of the pelvic girdle and hindlimb skeleton of UAMES 23258, holotype specimen of *Gunakadeit joseeae* (in mm). All measurements taken from the right side. *denotes estimated value.

Ilium length	22.5
Pubis length	18.0
Sacral rib length	17.0
Hindlimb length*	77.0
Pes length*	38.0
Manus width at metacarpals*	13.0
Femur anteroposterior length	26.0
Femur maximum proximal width	10.0
Femur maximum distal width	14.5
Tibia length	13.0
Tibia maximum width	7.5
Fibula length	13.5
Fibula proximal width	6.0
Fibula distal width	8.0

Supplementary Note 1: Institutional Abbreviations

SMNS, Staatliches Museum für Naturkunde Stuttgart, Germany; **TMP**; Royal Tyrrell Museum of Palaeontology, Drumheller Alberta, Canada; **UAMES**, University of Alaska Museum, Fairbanks, USA.

Supplementary Note 2: Phylogenetic Analysis

1. Character List

Abbreviations: N = Nicholls (1999)[1]; L = Liu and Rieppel (2005)[2]; M = Mueller (2007)[3]; LEA = Liu et al. (2013)[4]

CRANIUM

<u>**Skull roof**</u>

1. Rostral deflection: in lateral view, the dorsal margin of the rostrum (or dorsal margin of premaxilla, if disarticulated) is straight (0); dorsal margin of rostrum moderately deflected ventrally (>5-30 degrees) (1); strongly deflected (>30 degrees) (2). [M2, M3]

2. Rostral length: ratio of preorbital length to total skull length (measured along dorsal skull midline or to occipital condyle): <0.5 (0); 0.5-0.6 (1); >0.6 (2). [M40]

3. Position of external naris: at anterior end of rostrum (0); retracted posteriorly (1). [new character]

4. Anterior end of rostrum: acuminate (0), blunt and rounded (1). [L2]

5. Relative length of the rostrum and lower jaw: approximately equal (0); rostrum extends anterior of anterior end of dentary, producing a pronounced and distinct overbite (1). [new

character]

6. Alveolar margin (premaxilla and maxilla) of rostrum in lateral view: straight along its length (0); broadly embayed dorsally (1); abruptly embayed dorsally producing a distinct notch (2). [new character]

7. Posteroventral process of premaxilla: absent, premaxilla does not extend ventrally beyond anterior margin of naris (0); present, premaxilla extends ventrally beyond anterior margin of naris (1). [L5]

8. Relative length of premaxilla; anteroposteriorly short: less than half rostrum length (0); elongate, extending at least half the length of the rostrum (1). [N17]

9. Premaxilla-frontal contact; present (0); absent (1). [New character]

10. Proportions of maxilla: at least twice as anteroposteriorly long as dorsoventrally high (0); less than twice as long as high (1). [M4]

11. Posterodorsal process of the maxilla: a distinct and narrow, dorsoventrally extending process present (0); absent (1). [New character]

12. Alveolar margin of anterior process of maxilla; in lateral view, alveolar margin of maxilla is straight (0); margin is angled dorsally (1); margin is angled ventrally (2). [N10]

13. Maxilla participation in anterior margin of orbit: maxilla contributes substantially to anteroventral margin of orbit (0); maxilla does not or only barely participates in orbital margin (1). [New character]

14. Nasals contact along the dorsal midline; present (0); absent (1). [M13]

15. Nasal participation in dorsal border of external naris; nasal forms less than half of the dorsal border of the external naris, excluded by premaxilla (0); nasal forms most of dorsal border with little or no participation by premaxilla (1). [L9, M14]

16. Relative size of external naris and orbit (maximum anteroposterior dimensions); external naris small, <40 percent orbit length (0); external naris large, >40 percent orbit length (1). [New character]

17. Nasal-prefrontal contact: present (0); absent (1). [N14, L11, M17]

18. Posterior process of nasal: elongate, extending at least to the level of the anterior margin of the orbit (0); moderate, not reaching the anterior margin of the orbit (1); absent (2). [New character]

19. Prefrontal participates in the posterior border of the external naris; absent (0); present (1). [New character]

20. Prefrontal morphology: not laterally expanded, anterodorsal margin of orbit lacking a brow-like protrusion or corresponding recess (0); laterally expanded, forming a prominent "brow" along anterodorsal margin of orbit and corresponding ventral recess (1). [New character].

21. Lacrimal; present (0); absent (1). [N12]

22. Anterior extent of jugal; extends anterior to the anterior margin of the orbit (0); does not extend anterior of orbital margin (1). [New character]

23. Relative lengths of the anterior and posterior processes of the jugal; length of anterior

process approximately as long as posterior process (0); anterior process significantly longer than posterior process (1). [New character]

24. Posterolateral process of frontal; poorly developed or absent and not extending posterior of a point in line with the pineal foramen (0); well developed and extending posterior to the pineal foramen (1). [L14, M19]

25. Frontal-supratemporal contact: absent (0); present (1). [L13, M20]

26. Frontoparietal suture (excluding posterolateral process of frontal) in dorsal view: oriented transversely near the midline (0); embayed in the shape of a broad V, with the apex pointing anteriorly (1). [L15, M21]

27. Postfrontal and postorbital; unfused and represented as distinct ossifications (0); fused into a single element, the postorbitofrontal (1). [N16, L16, M22]

28. Shape of posterior skull margin in dorsal view: transversely straight, such that the posterior midline of the skull roof is approximately in line with the posterior margin of the squamosal and/or supratemporal (0); deeply embayed, such that the posterior midline of the skull lies far anterior to the posterior margin of the squamosal and/or supratemporal (1). [New character]

29. Upper temporal fenestra: present and large, comparable in size to lower temporal opening (0); present but much smaller than lower temporal opening and anteroposteriorly elongate ("slit-like") (1); absent (2). [M23]

30. Squamosal, posteroventral process: present (0); absent (1). [L17, M24]

31. Quadrate, expanded medial lamina (=anterior flange): present and significantly expanded

(0); absent or weakly developed (1). [M25]

32. Quadratojugal: present (0); absent (1). [M26]

33. Position of pineal foramen relative to postorbital bar: a transverse line drawn through the midpoint of pineal foramen lies posterior to the postorbital bar (0); lies in line with the postorbital bar (1); lies anterior to the postorbital bar (2). [L19, M27]

34. Size of pineal foramen relative to external naris: pineal foramen as large or larger than external naris (0); pineal foramen clearly smaller than external naris (1). [New character]

<u>Palate</u>

35. Vomerine dentition: present (0); absent (1). [N27, M12]

36. Pterygoid dentition: present (0); absent (1). [N25, M10, 11]

37. Fusion of vomers: Paired vomers fused anteriorly (0); paired vomers retain distinct suture along their entire contact, not fused anteriorly (1). [N20]

<u>Mandible</u>

38. Dorsal margin of anterior end of dentary: straight (0); ventrally deflected (1). [L20, 24, M8]

39. Length of mandibular symphysis: short and including ~4 or fewer tooth positions adjacent to the symphysis (0); long with >5 tooth positions adjacent to symphysis (1). [new character]

40. New character: splenial participates in mandibular symphysis: present (0); absent (1). [new character]

41. Dentary, posteroventral process on lateral surface: present (0); absent or very weakly developed (1). [LEA]

42. Anterior extent of dentary-angular suture in lateral view of mandibular ramus: does not extend anterior of the anterior margin of the orbit (0); clearly extends beyond the anterior margin of the orbit (1). [new character]

43. Coronoid eminence (of either the coronoid or surangular); tall and triangular or anteroposteriorly narrow (0); low and broadly rounded (1). [new character]

44. Retroarticular process: absent (0); present (1). [new character]

45. Retroarticular process: ventrally deflected (0), horizontal (1). [new character]

<u>Dentition</u>

46. Marginal dentition homodont (0); heterodont (1) (edentulous = "?"). [new character]

47. Premaxillary dentition: absent (0); present (1). [N29, M6, L22]

48. Extent of premaxillary dentition: toothrow incomplete, does not extend to anterior end of premaxilla (0); toothrow complete with teeth extending continuously to anterior end of premaxilla (1). (premaxillary teeth absent = ?) [new character]

49. Pseudodont projections on premaxilla: absent (0); present (1). [N29, M6, L22]

50. Diastema in upper toothrow near the premaxillary-maxillary suture: absent (0); present (1). [N28, L23, M7]

51. Posterior maxillary or posterior dentary dentition; conical with distinctly pointed apex (0); conical but robust with expanded base and rounded apex (1) relatively bulbous and blunt (2). (maxillary dentition absent = ?) [L25, M9]

52. Presence of caniniform dentition in upper or lower toothrow: absent (0); present (1). [new character]

POSTCRANIUM

Axial skeleton

53. Number of cervical vertebrae: <5 (0); 6-10 (1); >11 (2). [N35, L27, M29]

54. Number of dorsal vertebrae: 20 or less (0); 21–30 (1); 31 or more (2) [new character]

55. Number of sacral vertebrae: 2 (0); 3 (1), 4 or more (2). [new character]

56. Postaxial cervical vertebrae, height of neural spine: dorsoventrally short, height of neural spine less than or nearly equal to anteroposterior length (0); dorsoventrally tall, height equal to or greater than anteroposterior length (1). [L28, M30]

57. Dorsal vertebrae, dorsoventral height of neural spine relative to centrum height: neural spine approximately equal to, or only slightly greater, than centrum height (0); neural spine conspicuously taller (~1.5x) than centrum height (1). [new character]

58. Dorsal vertebrae, shape of neural spine in lateral view; greatest anteroposterior width occurs at, or ventral to, mid-height of spine (outline rectangular) (0); conspicuously wider in dorsal half of spine (outline an inverted trapezoid) (1). [new character]

59. Anterior caudal vertebrae, height of proximal neural spine; height of spine less than 3x anteroposterior length (0); spine very tall, typically at least 3x anteroposterior length (1). [L29, M31]

60. Anterior processes on cervical ribs: absent (0); present (1). [M32]

61. Sacral rib morphology; longest sacral rib relatively short and broad, with length of distal expansion more than half of total rib length (0); longest rib elongate, with distal expansion less than half of total rib length (1). [new character]

62. Gastralia; gastralia robust, approaching dorsal ribs in diameter (0); extremely thin and hair-like, less than one-fifth diameter of dorsal ribs (1); neither robust nor hair-like, approximately one-third to one-half rib diameter (2). [new character]

<u>Appendicular skeleton</u>

63. Interclavicle, shape of anterior end: cruciform, with distinct anterior process (0); anterior process reduced or absent, anterior end broadly rounded or straight (1). [new character]

64. Interclavicle, length of single lateral process: short, mediolateral width of lateral process less than twice the width of the proximal end of the posterior process (0); long, lateral process more than twice the width of the posterior process (1). [new character]

65. Clavicle, length relative to interclavicle; clavicle significantly shorter than anteroposterior length of interclavicle (0); clavicle approximately as long or longer than interclavicle (1). [new character]

66. Scapula: slender (0); broad, width approximately equal to height (1). [L30, M33]

67. Scapular anterior margin: approximately straight or slightly convex (0); strongly convex (1). [LEA]

68. Coracoid morphology: anteroposteriorly short (shorter than scapula) and dorsoventrally thick (0); anteroposteriorly long (as long or longer than scapula) and dorsoventrally thin (1). [New character]

69. Humerus, relative width of proximal and distal ends: width of proximal end of humerus less than or equal to distal end (0); proximal end wider than distal end (1). [LEA]

70. Humerus, deltopectoral crest: present and well developed (0); very reduced or absent (1). [N38, L31, M38]

71. Radius morphology: dumb-bell shaped, elongate and with distinct shaft throughout diaphysis (0); hatchet-shaped, distal end expanded preaxially (1); reniform, element expanded preaxially along most or all of its length (2). [L32, M35, 36]

72. Ilium morphology: ilium with distinct, horizontally oriented posterodorsal process set off from the rest of the ilium at a pronounced bend (0); ilium lacking a distinct posterodorsal process; the dorsal portion more vertically oriented and broadly expanded (1). [N39]

73. Pubis, obturator foramen: present (0); absent (1). [New character]

74. Femur, relative width of proximal and distal ends: proximal end approximately equal in width to distal end (0); proximal end much narrower than distal end (1). [L33]

75. Tibia morphology: dumb-bell shaped, with distinct shaft (0); expanded preaxially along most or all of its length (1). [New character]

76. Fibula, distal expansion: fibula not expanded distally (0); distal end expanded but not more than 2x proximal end (1); distal end expanded at least 2x proximal end (2). [L34, M39]

77. Relative lengths of femur and tibia: Tibia elongate, length nearly equal to femur length (tibia length >75% femur length) (0); tibia short, <75% femur length (1). [New character]

78. Phalangeal proportions: phalanges short with expanded proximal and distal articular ends (0); phalanges elongate, columnar in appearance and lacking conspicuously expanded articular ends (1). [New character]

2. Taxon-Character data matrix scores

Claudiosaurus germaini
00010 01010 1000? 00100 01100 00000 10000 01001 1010? 01100 00100 00001 0210? 11?0? 00000 000

Petrolacosaurus kansensis
00010 00010 10100 00100 01100 00000 00010 01001 1010? 01100 01100 00001 0?001 11000 00000 000

Youngina capensis
01010 00010 10100 00100 01110 00000 10000 010?? 10?0? 01100 00000 0000? 0?11? 11?0? 00?00 000

Agkistrognathus campbelli
????? ????? ????? ????? ????? ????? ????0 ??100 0???? 11101 11??? ????? ????? ????? ????? ???

Anshunsaurus huangguoshuensis
02110 00100 ?0?11 00000 ?1000 01120 1?001 1?010 1011? 01100 00210 0010? 00010 00100 01000 210

Anshunsaurus huangnihensis
??11? ??10? 1?011 00000 1??10 00120 ??00? ??01? ???0? 0???0 ??2?? 0???1 ????? 11100 01?00 110

Anshunsaurus wushaensis
01110 01100 ?0?11 0?1?0 110?? ?1?2? ?100? ??01? 00110 01100 00210 00101 ?000? 0?10? 0?000 210

Askeptosaurus italicus
02110 00100 10110 01000 01100 00110 11011 10001 0000? 11100 00210 00001 02011 11101 00000 110

Clarazia schinzi
11111 ??101 ??111 1?10? ?0111 1112? ?1010 001?1 01010 11101 20110 1??10 01011 011?? 10011 110

Concavispina biseridens
??1?? 2???0 111?1 00?11 1???? ?1??0 01??1 0???? 01010 11??? 2?021 1011? 02001 01011 20110 111

Endennasaurus acutirostris
02100 00100 00?10 0???0 ???1? ?11?? 1???1 11011 ???0? ?0?00 ?0200 00100 10110 11100 00000 110

Gunakadeit joseeae
02100 00100 00111 10201 1010? 11011 0121? ??01? 11111 01?0? 00011 11010 11?11 0?011 20111 111

Hescheleria rubeli
2?111 11101 00111 ???0? ??1?? ?1??? ????? ??1?? 01110 11101 10112 10111 ?10?1 0110? 10000 110

Koessen thalattosaur
????? ????? ????? ????? ????? ????? ????? ????? ????? ????? ????? ???1? ?1??? ????? 2???0 110

Miodentosaurus brevis
01110 00100 1?001 00000 11110 ?1120 ?1101 11?00 00111 01100 00210 00101 12100 11101 01000 210

Nectosaurus halius
2?1?1 ?1100 01??? ????1 11111 11?21 ?1??0 001?? 1?011 1???? 11??? ?111? ?1??? ????? 2??1? 11?

Oregon thalattosaur
2211? 10100 12011 10010 11101 11121 11210 00??? ????? 01101 10??? ?101? ???0? 11001 21?11 1??

Paralonectes merriami
1?1?1 21??0 01110 0110? 111?? ????0 ?1??0 0?10? ??110 11101 20??? ??1?? ????? ????? ????? ???

Thalattosaurus alexandrae
12110 11100 01111 11101 01111 1112? 01110 00101 1101? 10?11 21??? 11000 ????? 00010 2001? 11?

Thalattosaurus borealis
1?11? ?1100 0??11 ?110? 1???? ????? ????0 0???? 0???? 10?1? 2???? ????? ????? ????? ????? ???

TMP 88.99.21

????? ????? ????? ????? ????? ????? ????? ????? ????? ????? ????0 ?100? 0???? ????? ??000 111

Xinpusaurus kohi

01101 2?1?0 ?10?? 0?101 ?1111 1112? ??11? 0???? 01010 1100? 21110 1101? 12??? ???1? 2???0 2?1

Xinpusaurus suni

01111 21100 11011 01101 11?00 1112? 01?10 0?1?? 11010 11001 2112? 110?? ????? 0011? 2??1? 2??

Xinpusaurus xingyiensis

0?11? 2?1?0 ?10?? ????? ?11?? ????? 0???0 000?? 11010 1100? ??0?? 0001? ?200? 01110 20000 111

3. Analysis and results

Iteration 1: Analysis of the full matrix of 23 taxa and 78 characters.

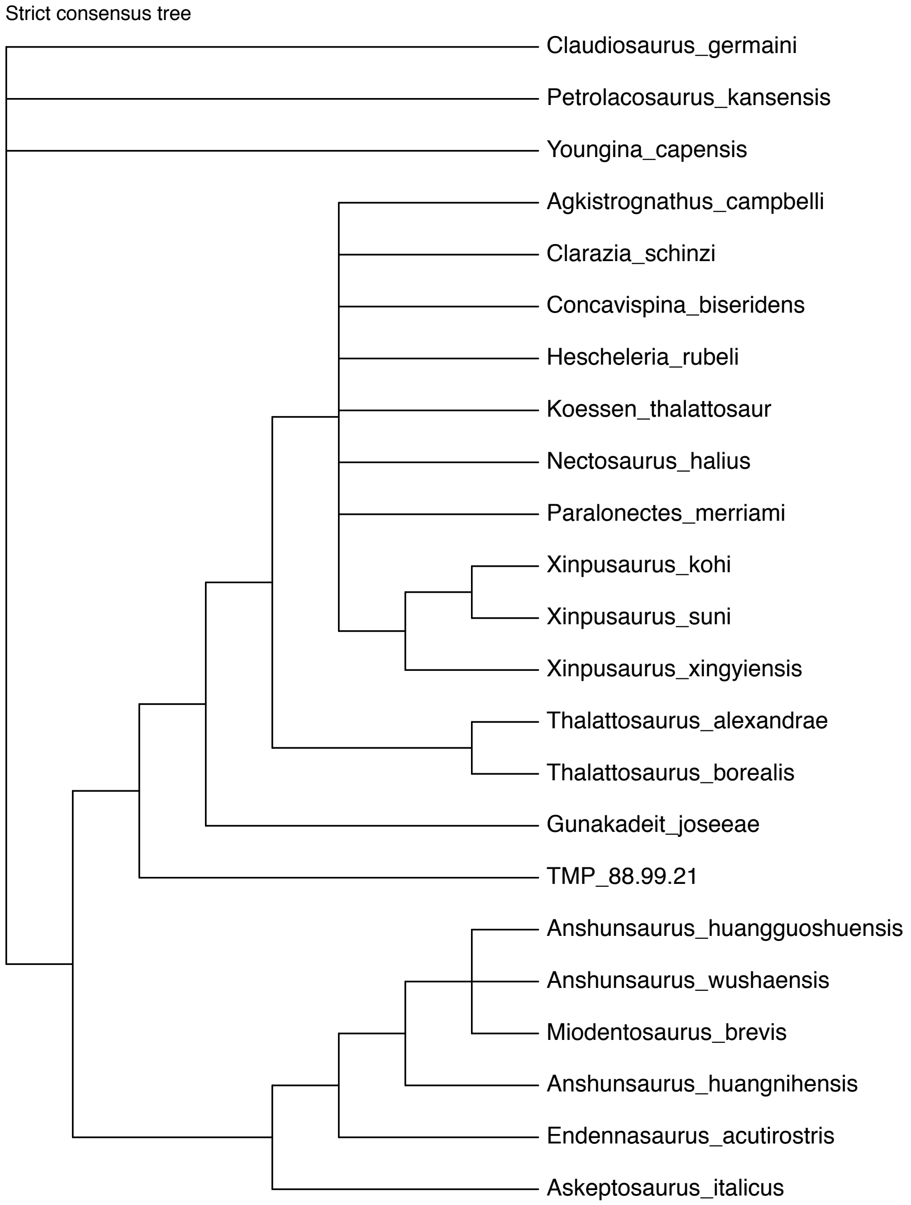

Supplementary Figure 1. Strict consensus tree (of 44 trees at tree length of 192 steps) of the full matrix (CI=0.474; RI= 0.668).

Iteration 2: Analysis after removal of 3 taxa (*Agkistrognathus*, *Paralonectes* and TMP 88.99.21).

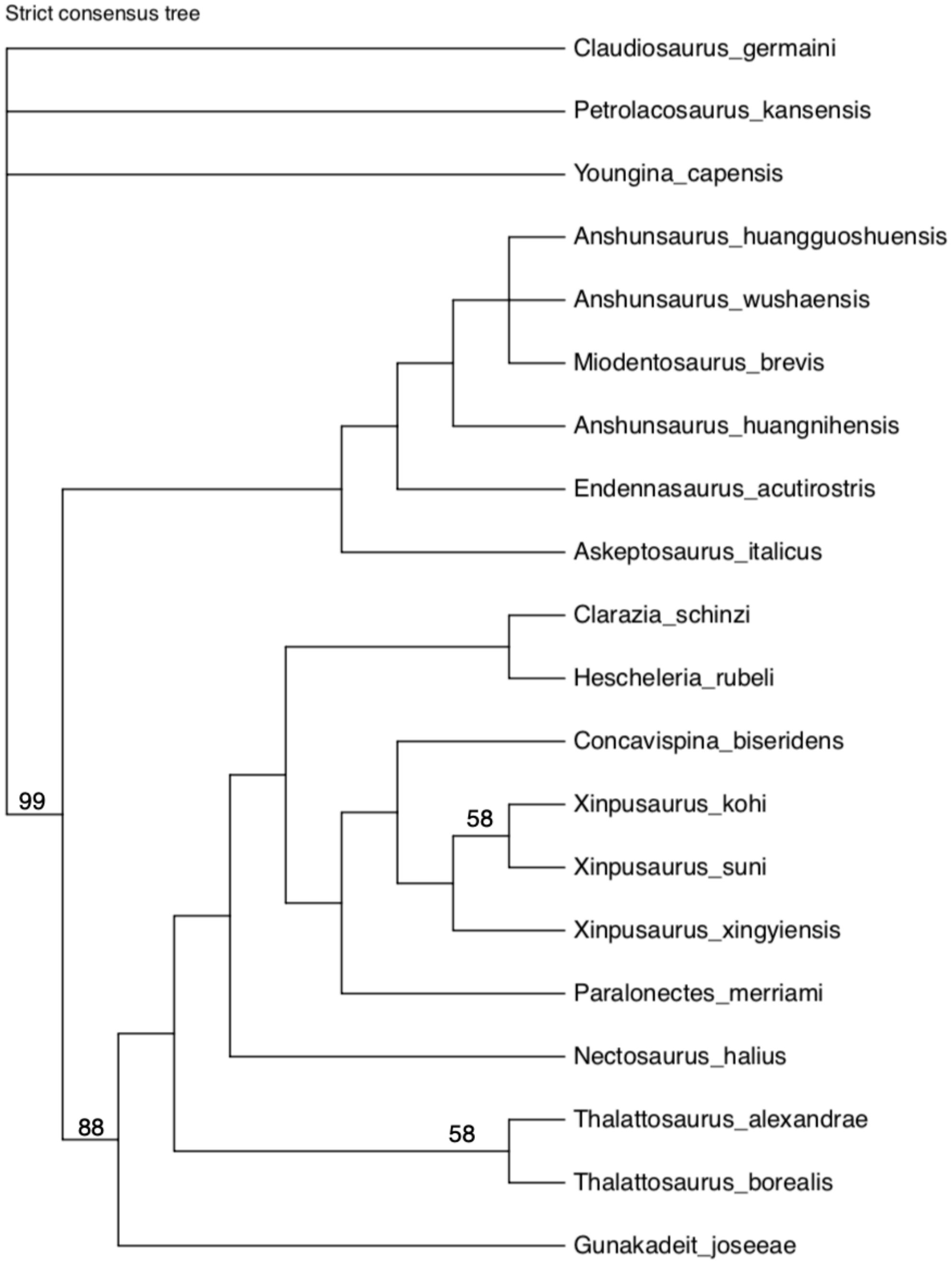

Supplementary Figure 2. Strict consensus tree (of 4 trees at tree length of 190 steps) of the full matrix (CI=0.479; RI=0.662). Bootstrap percentages for nodes greater than 50% shown above the branches.

Supplementary References

1. Nicholls, E. L. A reexamination of *Thalattosaurus* and *Nectosaurus* and the relationships of the Thalattosauria (Reptilia: Diapsida). *PaleoBios* **19**, 1–29 (1999).
2. Liu, J. & Rieppel, O. Restudy of *Anshunsaurus huangguoshuensis* (Reptilia: Thalattosauria) from the Middle Triassic of Guizhou, China. *Am. Museum Novit.* **3488**, 1–34 (2005).
3. Müller, J. First record of a thalattosaur from the upper Triassic of Austria. *J. Vertebr. Paleontol.* **27**, 236–240 (2007).
4. Liu, J., Zhao, L.-J., Li, C. & He, T. Osteology of *Concavispina biseridens* (Reptilia, Thalattosauria) from the Xiaowa Formation (Carnian), Guanling, Guizhou, China. *J. Paleontol.* **87**, 341–350 (2013).
