# Supplementary material for: An articulated Late Triassic (Norian) thalattosauroid from Alaska and ecomorphology and extinction of Thalattosauria
Source: Sci Rep. 2020 Feb 4;10:1746. doi: 10.1038/s41598-020-57939-2 (PMC7000825; doi:10.1038/s41598-020-57939-2)
Supplement: Supplementary file 2 — Supplementary Dataset 1. [file 41598_2020_57939_MOESM2_ESM.pdf]

Supplementary Data

An articulated Late Triassic (Norian) thalattosauroid from Alaska and ecomorphology and extinction of Thalattosauria

Patrick S. Druckenmiller, Neil P. Kelley, Eric T. Metz and James Baichtal

```
#NEXUS
[written Wed Sep 25 11:57:08 AKDT 2019 by Mesquite  version 3.6 (build
917) at FBK-021J1GN.local/172.20.154.63]

BEGIN TAXA;
      TITLE Taxa;
      DIMENSIONS NTAX=24;
      TAXLABELS
            'Claudiosaurus_germaini' 'Petrolacosaurus_kansensis'
'Youngina_capensis' 'Agkistrognathus_campbelli'
'Anshunsaurus_huangguoshuensis' 'Anshunsaurus_huangnihensis'
'Anshunsaurus_wushaensis' 'Askeptosaurus_italicus' 'Clarazia_schinzi '
'Concavispina_biseridens' 'Endennasaurus_acutirostris'
'Gunakadeit_joseeae' 'Hescheleria_rubeli' 'Koessen_thalattosaur'
'Miodentosaurus_brevis' 'Nectosaurus_halius' 'Oregon_thalattosaur'
'Paralonectes_merriami' 'Thalattosaurus_alexandrae '
'Thalattosaurus_borealis' 'TMP_88.99.21' 'Xinpusaurus_kohi '
'Xinpusaurus_suni' 'Xinpusaurus_xingyiensis'
      ;

END;


BEGIN CHARACTERS;
      TITLE  Character_Matrix;
      DIMENSIONS  NCHAR=78;
      FORMAT DATATYPE = STANDARD RESPECTCASE GAP = - MISSING = ? SYMBOLS
= "  0 1 2";
      CHARSTATELABELS
            1 #1,
            2 #2,
            3 #3,
            4 #4,
            5 #5,
            6 #6,
            7 #7,
            8 #8,
            9 #9,
            10 #10,
            11 #11,
            12 #12,
            13 #13,
            14 #14,
            15 #15,
            16 #16,
            17 #17,
            18 #18,
            19 #19,
            20 #20,
            21 #21,
            22 #22,
            23 #23,
            24 #24,
            25 #25,
```

```
        26 #26,
        27 #27,
        28 #28,
        29 #29,
        30 #30,
        31 #31,
        32 #32,
        33 #33,
        34 #34,
        35 #35,
        36 #36,
        37 #37,
        38 #38,
        39 #39,
        40 #40,
        41 #41,
        42 #42,
        43 #43,
        44 #44,
        45 #45,
        46 #46,
        47 #47,
        48 #48,
        49 #49,
        50 #50,
        51 #51,
        52 #52,
        53 #53,
        54 #54,
        55 #55,
        56 #56,
        57 #57,
        58 #58,
        59 #59,
        60 #60,
        61 #61,
        62 #62,
        63 #63,
        64 #64,
        65 #65,
        66 #66,
        67 #67,
        68 #68,
        69 #69,
        70 #70,
        71 #71,
        72 #72,
        73 #73,
        74 #74,
        75 #75,
        76 #76,
        77 #77,
        78 #78 ;
MATRIX
```

'Claudiosaurus_germaini'
000100101010000?001000110000000010000010011010?0110000100000010210?11?0?000
00000
'Petrolacosaurus_kansensis'
0001000010101000010001100000000000100100110 10?01100011000000 10?00111000000
00000
'Youngina_capensis'
0101000010101000010001110000000100000 10??10?0?0110000000000?0?11?11?0?00?
00000
'Agkistrognathus_campbelli'
??????????????????????????????????0??1000????110111?????????????????????
?????
'Anshunsaurus_huangguoshuensis'0211000100?0?1100000?1000011201?0011
?0101011?01100002100010?000100010001000210
'Anshunsaurus_huangnihensis'
??11???10?1?011000001??1000120??00???01????0?0???0??2??0???1?????1110001?
00110
'Anshunsaurus_wushaensis'
011100110 0?0?110?1?0110???1?2??100???01?00110011000021000101?000?0?10?0?0
00210
'Askeptosaurus_italicus'
02110001001011001000011000011011011100010000?11100002100000102011110 1000
00110
'Clarazia_schinzi '
11111??101??1111?10??0111111 2??1010001?1010101110120110 1??1001011011??100
11110
'Concavispina_biseridens'
??1??2???0111?100?111?????1??001??10???010101 1???2?0211011?0200101011201
10111
'Endennasaurus_acutirostris'
02100001000 0?100???0???1??11??1???111011???0??0?00?0200001001011011100000
00110
'Gunakadeit_joseeae'
0210000100001111 02011010?110110121???01?1111101?0?0001111 01011?110?011201
11111
'Hescheleria_rubeli'
2?1111110100111???0???1???1??????????1??011101110110112 10111?10?10110?100
00110
'Koessen_thalattosaur'
??1??1????????2??
?0110
'Miodentosaurus_brevis'
0111000100 1?001000000111 10?1120?110111?000011101100002100010112100111010 10
00210
'Nectosaurus_halius'
2?1?1?110001??????1111111?21?1??0001??1?0111?????11????111??1????????2??
1?11?
'Oregon_thalattosaur'
2211?1010012011100101110111121112 1000???????0110110????101????0?1100121?
111??
'Paralonectes_merriami'
1?1?121??0011100110?111??????0?1??00?10???1101110120?????1???????????????
?????

```
	'Thalattosaurus_alexandrae '
12110111000111111101011111112?01110001011101?10?1121???11000?????00010200
1?11?
	'Thalattosaurus_borealis'
1?11??11000??11?110?1?????????????00????0????10?1?2??????????????????????
?????
	'TMP_88.99.21'
??????????????????????????????????????????????????????0?100?0???????????0
00111
	'Xinpusaurus_kohi '
011012?1?0?10??0?101?11111112???11?0????010101100?211101101?12??????1?2??
?02?1
	'Xinpusaurus_suni'
0111121100110110110111?001112?01?100?1??11010110012112?110???????0011?2??
1?2??
	'Xinpusaurus_xingyiensis'
0?11?2?1?0?10????????11???????0???0000??11010110 0???0??0001??200?01110200
00111

;

END;
BEGIN ASSUMPTIONS;
	TYPESET * UNTITLED   =  unord:  1- 78;

END;

BEGIN MESQUITECHARMODELS;
	ProbModelSet * UNTITLED   =  'Mk1 (est.)':  1- 78;
END;

Begin MESQUITE;
		MESQUITESCRIPTVERSION 2;
		TITLE AUTO;
		tell ProjectCoordinator;
		timeSaved 1569441428989;
		getEmployee #mesquite.minimal.ManageTaxa.ManageTaxa;
		tell It;
			setID 0 5384719305773318 91;
		endTell;
		getEmployee 
#mesquite.charMatrices.ManageCharacters.ManageCharacters;
		tell It;
			setID 0 8571457993383312595;
			mqVersion 360;
			checksumv 0 3 555636633 null  getNumChars 78 numChars 78 
getNumTaxa 24 numTaxa 24   short true   bits 7   states 7 
sumSquaresStatesOnly 3923.0 sumSquares 3923.0 longCompressibleToShort 
false usingShortMatrix true   NumFiles 1 NumMatrices 1;
			mqVersion;
		endTell;
		getWindow;
		tell It;
			suppress;
```

```
				setResourcesState false false 159;
				setPopoutState 300;
				setExplanationSize 0;
				setAnnotationSize 0;
				setFontIncAnnot 0;
				setFontIncExp 0;
				setSize 2323 981;
				setLocation 8 23;
				setFont SanSerif;
				setFontSize 10;
				getToolPalette;
				tell It;
				endTell;
				desuppress;
			endTell;
			getEmployee
#mesquite.charMatrices.BasicDataWindowCoord.BasicDataWindowCoord;
			tell It;
				showDataWindow #8571457993383312595
#mesquite.charMatrices.BasicDataWindowMaker.BasicDataWindowMaker;
				tell It;
					getWindow;
					tell It;
						getTable;
						tell It;
							rowNamesWidth 224;
						endTell;
						setExplanationSize 30;
						setAnnotationSize 20;
						setFontIncAnnot 0;
						setFontIncExp 0;
						setSize 2164 909;
						setLocation 8 23;
						setFont SanSerif;
						setFontSize 10;
						getToolPalette;
						tell It;
							setTool
mesquite.charMatrices.BasicDataWindowMaker.BasicDataWindow.arrow;
						endTell;
						setActive;
						setTool
mesquite.charMatrices.BasicDataWindowMaker.BasicDataWindow.arrow;
						colorCells
#mesquite.charMatrices.NoColor.NoColor;
						colorRowNames
#mesquite.charMatrices.TaxonGroupColor.TaxonGroupColor;
						colorColumnNames
#mesquite.charMatrices.CharGroupColor.CharGroupColor;
						colorText
#mesquite.charMatrices.NoColor.NoColor;
						setBackground White;
						toggleShowNames on;
						toggleShowTaxonNames on;
```

```
					toggleTight off;
					toggleThinRows off;
					toggleShowChanges on;
					toggleSeparateLines off;
					toggleShowStates on;
					toggleAutoWCharNames on;
					toggleAutoTaxonNames off;
					toggleShowDefaultCharNames off;
					toggleConstrainCW on;
					toggleBirdsEye off;
					toggleShowPaleGrid off;
					toggleShowPaleCellColors off;
					toggleShowPaleExcluded off;
					togglePaleInapplicable on;
					togglePaleMissing off;
					toggleShowBoldCellText off;
					toggleAllowAutosize on;
					toggleColorsPanel off;
					toggleDiagonal on;
					setDiagonalHeight 80;
					toggleLinkedScrolling on;
					toggleScrollLinkedTables off;
				endTell;
				showWindow;
				getWindow;
				tell It;
					forceAutosize;
				endTell;
				getEmployee
#mesquite.charMatrices.AlterData.AlterData;
				tell It;
					toggleBySubmenus off;
				endTell;
				getEmployee
#mesquite.charMatrices.ColorByState.ColorByState;
				tell It;
					setStateLimit 9;
					toggleUniformMaximum on;
				endTell;
				getEmployee
#mesquite.charMatrices.ColorCells.ColorCells;
				tell It;
					setColor Red;
					removeColor off;
				endTell;
				getEmployee
#mesquite.categ.StateNamesStrip.StateNamesStrip;
				tell It;
					showStrip off;
				endTell;
				getEmployee
#mesquite.charMatrices.AnnotPanel.AnnotPanel;
				tell It;
					togglePanel off;
```

```
                        endTell;
                        getEmployee
#mesquite.charMatrices.CharReferenceStrip.CharReferenceStrip;
                        tell It;
                              showStrip off;
                        endTell;
                        getEmployee
#mesquite.charMatrices.QuickKeySelector.QuickKeySelector;
                        tell It;
                              autotabOff;
                        endTell;
                        getEmployee
#mesquite.charMatrices.SelSummaryStrip.SelSummaryStrip;
                        tell It;
                              showStrip off;
                        endTell;
                        getEmployee
#mesquite.categ.SmallStateNamesEditor.SmallStateNamesEditor;
                        tell It;
                              panelOpen true;
                        endTell;
                  endTell;
            endTell;
            endTell;
end;
```
